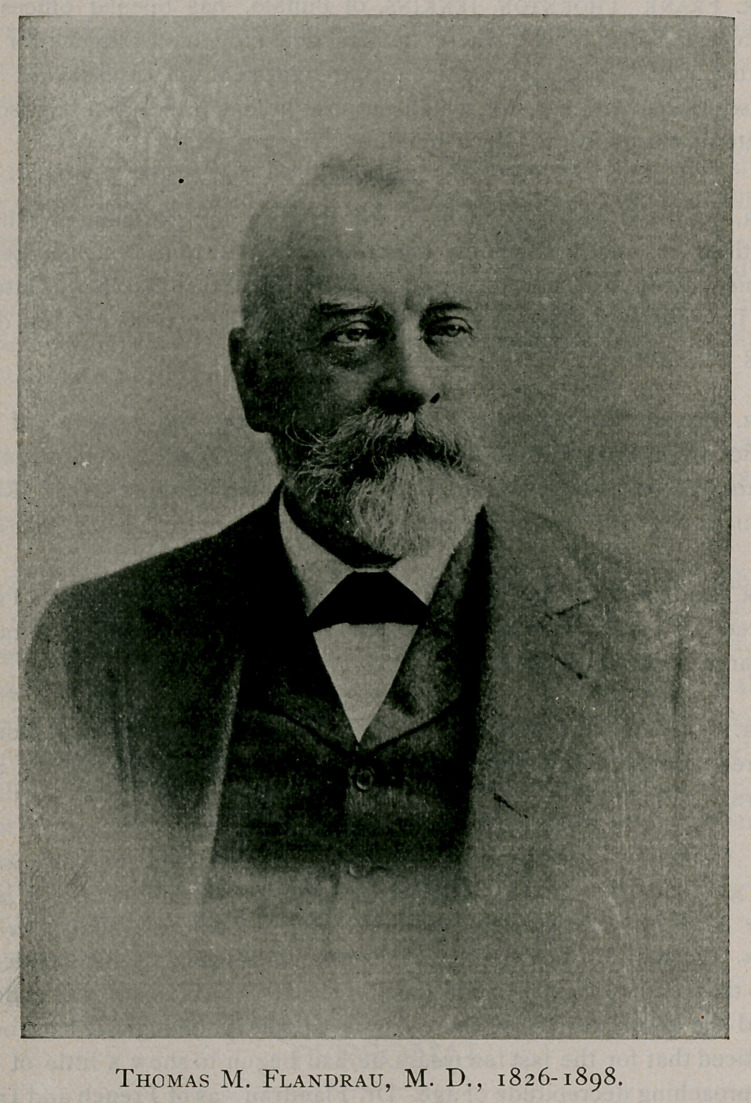# Dr. Thomas M. Flandrau

**Published:** 1898-09

**Authors:** 


### OBITUARY.

Dr. Thomas M. Flandrau, of Rome, N. Y., died at his residence in that city August 8, 1898, aged 72 years. His death was sudden and the immediate cause thereof was apoplexy, though it had been noticed that for the last few years he had begun to show a little of the approaching decrepitude of age. Dr. Flandrau was of French and Irish descent, the founder of the family in America being a French Huguenot who was driven from France in the time of Louis XIV. His mother was Elizabeth Macomb, daughter of Alexander Macomb, formerly of Detroit. Dr. Thomas Macomb Flandrau was born in

New York City, July 8, 1826. He passed his youth in Georgetown, D. C., and was educated in the private schools and academies of Georgetown and Washington. He studied medicine with Dr. Benjamin S. Bohrer and was graduated from the National Medical College,

THOMAS M. FLANDRAU, M. D., 1826-1898.

Washington, in March, 1848. He practised his profession a short time in Georgetown and then removed to his father's home in Whitesboro, Oneida county, but on January 1, 1853, he located at Rome, and for two years was associated with the late Dr. A. B. Blair in the practice of his profession. In 1856, he moved to Brockport, where he lived nearly six years, returning to Rome in 1862.

Dr. Flandrau was commissioned as surgeon of the 146th Regiment New York Volunteer Infantry, October 10, 1862, and his regiment in less than a month thereafter joined the Army of the Potomac and became a part of the third brigade, second division, fifth army corps. In June, 1864, he was appointed surgeon-in-chief of the division and upon the muster out of his regiment, July, 1865, he was brevetted lieutenant-colonel of United States Volunteers for faithful and meritorious service.

Dr. Flandrau's skill in surgery gave him prominence during his army life and the reputation then obtained lasted him to the end of his years. His wife died suddenly, May 1, 1890, though three children survive: Miss Elizabeth Flandrau and Mrs. H. C. Sutton, of Rome, and Mrs. George Ethridge, of New York. He also leaves one brother, Judge Charles Flandrau, a distinguished citizen of St. Paul, Minn. His son-in-law, Dr. H. C. Sutton, a prominent physician in Rome, was in attendance and ministered unto him in his last moments.

Dr. Flandrau was a man of many accomplishments, a graceful writer, a linguist, courtly in manner, kindly in disposition, skilful in his profession—easily the foremost physician in his region—and a citizen respected by the community in all the various walks of life. He was a member of the several medical societies—local, state, national and international,—and died lamented by a vast acquaintance.

## Figures and Tables

**Figure f1:**